# Plasma extrachromosomal circular DNA as a biomarker in EGFR‐targeted therapy of non‐small cell lung cancer

**DOI:** 10.1002/1878-0261.70138

**Published:** 2025-10-30

**Authors:** Simone Stensgaard, Sarmad Mehmood, Eva Boysen Fynboe Ebert, Peter Meldgaard, Anindya Dutta, Boe S. Sorensen

**Affiliations:** ^1^ Department of Clinical Biochemistry Aarhus University Hospital Denmark; ^2^ Department of Clinical Medicine Aarhus University Denmark; ^3^ Department of Genetics University of Alabama at Birmingham Alabama USA; ^4^ Department of Oncology Aarhus University Hospital Denmark

**Keywords:** cancer biomarkers, EGFR‐TKI, extrachromosomal circular DNA, liquid biopsy, lung cancer

## Abstract

The genomic instability associated with cancer can result in the formation of extrachromosomal circular DNA (eccDNA), which contributes to tumor heterogeneity, gene amplification, tumor evolution, and drug resistance. However, most studies on eccDNA have been conducted on tumor tissue or cancer cell lines, and limited research has been done on eccDNA in plasma. In this study, we investigated eccDNA in non‐small cell lung cancer (NSCLC) by sequencing plasma eccDNA from 32 epidermal growth factor receptor (*EGFR*)‐mutated NSCLC patients before and during treatment with osimertinib, as well as plasma eccDNA from five healthy individuals. Plasma eccDNA was identified in all samples but with significantly higher levels in cancer patients than healthy controls. *EGFR*‐overlapping eccDNA, eccDNA that contains part of or the whole *EGFR* gene, was detected in the majority of samples both at baseline and during treatment. High levels of *EGFR*‐overlapping eccDNA during osimertinib treatment were associated with significantly shorter progression‐free survival and overall survival. Plasma eccDNA represents a newly identified type of biomarker for monitoring treatment efficacy.

## Graphical abstract

Detection of extrachromosomal circular DNA (eccDNA) in plasma samples from *EGFR*‐mutated non‐small cell lung cancer patients. Plasma was collected before and during treatment with the EGFR‐tyrosine kinase inhibitor osimertinib. Plasma eccDNA was detected in all cancer samples, and the presence of the *EGFR* gene on eccDNA serves as a potential biomarker for treatment response.

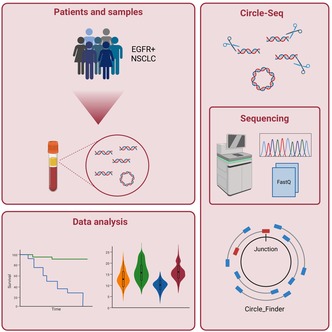

**Abbreviations**
eccDNA: extrachromosomal circular DNA
*EGFR*
: epidermal growth factor receptor
NSCLC: non‐small cell lung cancer
TKI: tyrosine kinase inhibitor

## 1 Introduction

An emerging topic in DNA research is the study of circular DNA fragments that reside outside of the chromosomes. Extrachromosomal circular DNA (eccDNA) is common in eukaryotic cells and includes mitochondrial DNA, telomeric circles, and 5S ribosomal DNA [1, 2]. Furthermore, eccDNA can arise due to DNA damage, chromothripsis, episomes, and large structural variations in the genome, resulting in the creation of double minutes and microDNA [2, 3, 4]. These events are especially common in cancer cells, and the presence of double minutes in tumor tissue was discovered in 1965 [5]. Their presence has since been shown in a large variety of cancer types [3, 6, 7, 8, 9]. However, eccDNA is not limited to tumor tissues, and studies have also demonstrated the presence of eccDNA in normal tissues [10, 11]. It has been proposed that large eccDNA in tumor tissues plays a crucial role in oncogene amplification, treatment resistance, immune response, and intercellular crosstalk [2, 3, 12, 13]. The role of smaller eccDNA is still largely unknown, but it has been proposed to have a gene regulatory function [14]. An important feature of eccDNA is that, because it resides outside the chromosomes and lacks centromeres, it is subjected to random segregation during mitosis, thereby contributing to tumor heterogeneity [9, 15].

Although most studies on eccDNA have been conducted in cancer cell lines or solid tumor tissue, eccDNA has also been identified in plasma from a wide range of sources, including healthy individuals, mice, pregnant women, cancer patients, and diabetic patients [16, 17, 18, 19, 20, 21, 22]. However, information regarding plasma eccDNA detection in a large homogeneous cohort of cancer patients is lacking, as is a study into the dynamics of plasma eccDNA in response to systemic treatment. One of the most successful targeted therapies in the treatment of lung cancer is the drug osimertinib, which is a third‐generation epidermal growth factor receptor (EGFR) tyrosine kinase inhibitor (TKI). Osimertinib has demonstrated an objective response rate of 80% as well as a prolonged progression‐free survival (PFS) and overall survival (OS) compared with earlier generations of EGFR‐TKIs [23]. Despite the promising response rate and survival outcomes, patients treated with osimertinib eventually develop resistance to treatment, and the resistance mechanisms are not yet fully understood. Studies have proposed that resistance to EGFR‐TKIs may be mediated by amplification of *EGFR* on eccDNA [12, 24].

In this study, we analyzed plasma eccDNA from a cohort of 32 patients with *EGFR*‐mutated non‐small cell lung cancer (NSCLC), with plasma samples collected both before and during treatment with osimertinib. Our aim was to characterize plasma‐derived eccDNA in NSCLC patients compared with healthy individuals. Furthermore, we investigated overlaps between eccDNA and the *EGFR* gene, the oncogenic driver in the patient cohort, to evaluate the potential of eccDNA as a biomarker for response to EGFR‐targeted therapy.

## 2 Materials and methods

### 2.1 Patients

Patients were included in this retrospective study from an observational prospective study, which enrolled *EGFR*‐mutated NSCLC patients from four Danish hospitals (Herning Hospital, Odense University Hospital, Aalborg University Hospital, and Aarhus University Hospital) from September 2014 to December 2018 (ClinicalTrials.gov ID: NCT02284633). The study was conducted in accordance with the Declaration of Helsinki, and all patients provided written informed consent. The study was approved by the Danish National Committee on Health Ethics (no.: 1‐10‐72‐83‐14) and the Danish Data Protection Agency (no.: 1‐16‐02‐431‐14).

Patients were included if they had received treatment with osimertinib as the first‐, second‐, or third‐line treatment. All patients receiving osimertinib as a second‐ or third‐line treatment had formerly been treated with erlotinib. Enrolled patients were required to have a plasma sample collected up to 2 weeks before the start of osimertinib treatment. This sample is referred to as the baseline sample. An additional response plasma sample was included for each patient if a sample had been collected during treatment. If several response plasma samples were available, the sample collected closest to treatment initiation was included. Furthermore, plasma samples from five healthy individuals were included as healthy controls. The state of disease was assessed using computed tomography (CT) imaging in accordance with the RECIST criteria. In cases where a CT scan was not performed at the time of response sample collection, the CT scan conducted closest to the collection date was utilized for evaluation.

### 2.2 Sample collection and eccDNA enrichment

Peripheral blood samples were collected in ethylenediaminetetraacetic acid (EDTA) tubes. The blood samples were centrifuged at 1400 ***g*** for 15 min, and plasma was aliquoted and stored at −80 °C. Cell‐free DNA (cfDNA) was isolated from a median of 3.8 mL plasma (1.8–4.6 mL) using the AVENIO cfDNA Isolation Kit (Roche, Basel, Switzerland), and it was eluted in 50 μL elution buffer for cancer samples and 100 μL elution buffer for healthy controls. The DNA concentration was measured using the Qubit dsDNA HS assay kit (Thermo Fischer Scientific, Waltham, MA, USA). Mitochondrial DNA was cleaved using the MssI (PmeI) restriction enzyme (Thermo Fischer Scientific). The cfDNA was treated with MssI for 16 h at 37 °C, and the enzyme was deactivated at 65 °C for 20 min. Afterward, the linear DNA was removed using Plasmid‐Safe ATP‐Dependent DNase (LGC Biosearch Technologies, Hoddesdon, United Kingdom) at 37 °C for 30 min, followed by inactivation of the enzyme at 70 °C for 30 min. The resulting eccDNA was cleaned using ethanol precipitation. The eccDNA was subjected to multiple displacement amplification using the REPLI‐g Single Cell Kit (QIAGEN, Hilden, Germany), followed by clean‐up via ethanol precipitation. The eccDNA was sonicated to a desired length of 100–500 bp using the Bioruptor Sonication System (Diagenode, Liège, Belgium). The final DNA concentration was quantified using the Qubit dsDNA HS assay kit.

### 2.3 Library preparation and next‐generation sequencing

Library preparation and next‐generation sequencing were performed by the NGS Core Center, Department of Molecular Medicine, Aarhus University Hospital, Denmark. The eccDNA‐enriched DNA library was prepared using the Kapa HyperPrep kit (Roche) and was subjected to 151‐bp paired‐end sequencing on a NovaSeq 6000 (Illumina, San Diego, CA, USA) with an expected depth of 30 million read pairs per sample.

### 2.4 Identification of plasma eccDNA

The quality of the fastq files was evaluated using fastqc [25] and the reads were trimmed using TrimGalore [26]. The eccDNA was identified using the Circle_Finder algorithm, which Shibata *et al*. [11] previously described. The Circle_Finder pipeline first maps the paired‐end reads onto the genome using the bwa‐mem aligner [27]. The algorithm then identifies split reads, which map to three sites in the genome, where one of the paired‐end reads maps contiguously and the other as a split read. If the contiguous mapped read is positioned between the two parts of the split read, as well as on the opposite strand, an eccDNA molecule is identified. The number of eccDNA molecules identified using Circle_Finder is referred to as the unfiltered eccDNA. A filter was afterward applied, which included only eccDNA mapped to chromosomes 1 to 22, X, and Y with at least two junctional tags, which is the number of reads mapping to the eccDNA breakpoint. Guanine‐cytosine (GC) content was calculated for every eccDNA, as well as for the regions directly upstream and downstream of the eccDNA corresponding to the size of the eccDNA. One thousand random fragments of the median size of the eccDNA were extracted from the hg38 assembly for comparison. Microhomology was calculated as the presence of direct repeats of 2‐ to 15‐bp upstream and downstream of the eccDNA breakpoint.

### 2.5 *EGFR* ‐overlapping eccDNA

eccDNA that contains part of or the whole *EGFR* gene is referred to as *EGFR*‐overlapping eccDNA. These molecules were identified by comparing the genomic position of each eccDNA molecule with the genomic position of *EGFR* (chromosome 7, 55 019 017–55 211 628). If an overlap between the positions of the eccDNA and *EGFR* was identified, the eccDNA was included in the *EGFR‐*overlapping eccDNA. The number of *EGFR*‐overlapping eccDNA was normalized to the total number of eccDNA identified in each patient.

### 2.6 Statistical analysis

Differences between the two groups were calculated using the Wilcoxon rank‐sum test. All tests were two‐tailed, and *P*‐values below 0.05 were considered significant. Data analysis and visualization were carried out in GraphPad Prism version 10.2.3 (graphPad Software, San Diego, CA, USA), r statistical Software version 4.2.1 (R Core Team, Vienna, Austria), and biorender (BioRender, Toronto, ON, Canada). The packages dplyr [28], ggbreak [29], ggplot2 [30], and ggpubr [31] were used for visualization in R Statistical Software. The enrichment analysis of genomic elements was conducted using the GenomicDistributions and GenomicDistributionsData packages [32], and the fold enrichment was calculated as the observed/expected number of eccDNA per genomic element using the hg38 assembly as a reference genome. Kaplan–Meier analysis was conducted using the survminer [33] and survival [34] packages, and the hazard ratio (HR) was calculated with the Cox proportional hazards model. PFS was defined as the time from osimertinib treatment initiation to the date of radiologically detected progression determined using the RECIST criteria, or death, whichever came first. If patients had not yet progressed, they were censored at their last follow‐up scan before the data cutoff of June 1, 2024. OS was calculated as the time from treatment initiation to the date of death. Patients without complete survival data were censored at the data cutoff date.

## 3 Results

### 3.1 Patients

Thirty‐two patients with *EGFR*‐mutated advanced NSCLC were included in the study. All patients had an available baseline plasma sample, collected before osimertinib treatment, and 30/32 (93.8%) had a response plasma sample, collected after a median of 28 days (range 21–90 days). During the study period, 27/32 patients (84.4%) died. Additionally, a total of 29/32 patients (90.6%) either experienced disease progression or died without previously detected disease progression. The majority of patients received osimertinib as second‐line treatment (81.3%), with three patients (9.4%) receiving osimertinib as first‐line treatment and three patients (9.4%) receiving osimertinib as third‐line treatment. All patients who received osimertinib as a second‐ or third‐line treatment had previously been treated with the first‐generation EGFR‐TKI erlotinib. Of the patients treated with erlotinib, 23/29 had progressive disease at the time of the collection of the baseline sample, whereas six patients had stable disease. The patients being treated with osimertinib as a third‐line treatment have, besides erlotinib, either received pembrolizumab or carboplatin and vinorelbine. The baseline clinical characteristics are listed in Table S1.

### 3.2 Characterization of eccDNA in baseline plasma samples

**Fig. 1:**
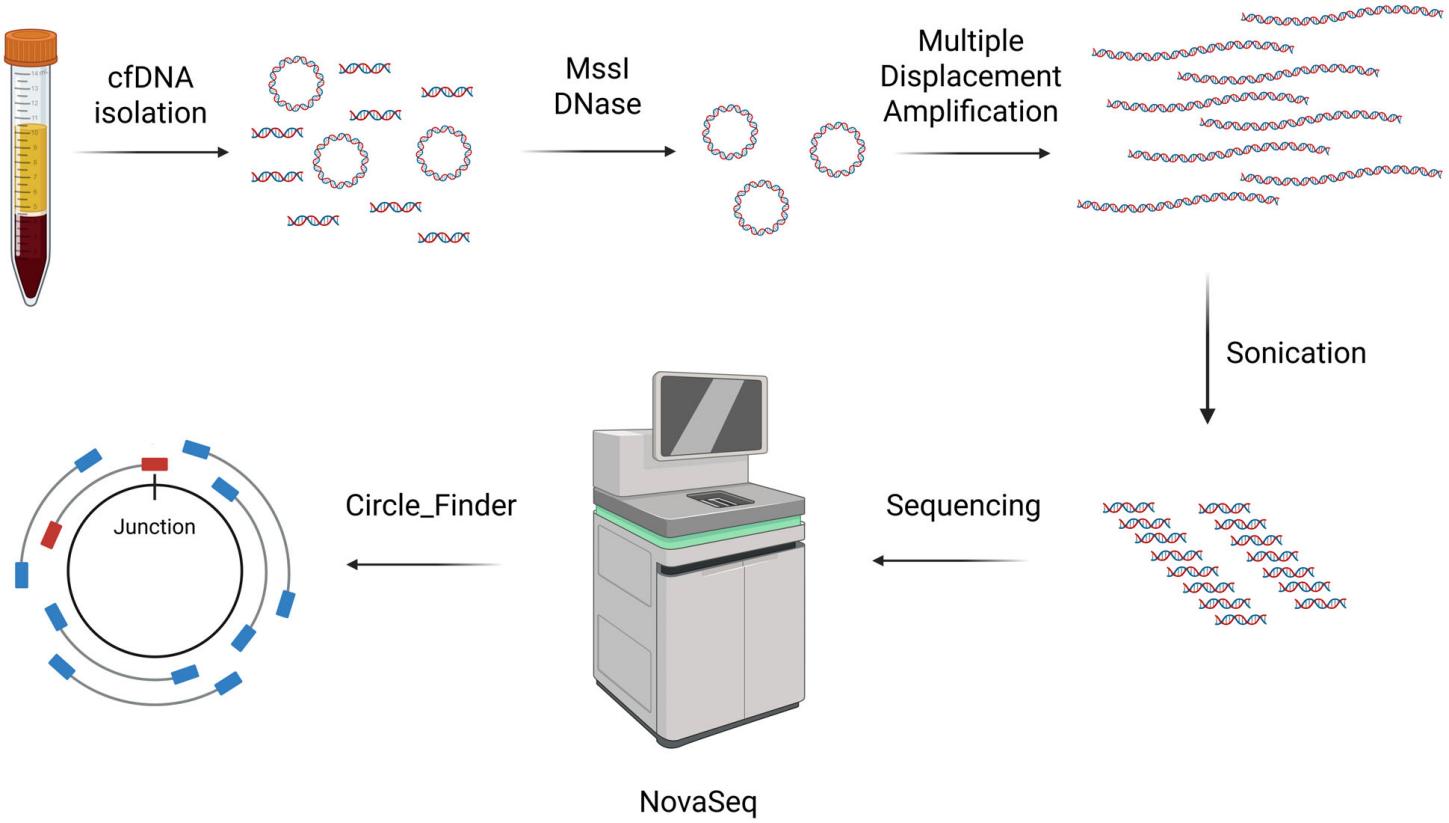
Workflow of extrachromosomal circular DNA (eccDNA) enrichment from plasma. cfDNA was isolated and subjected to digestion with MssI and an exonuclease to remove mitochondrial and linear DNA fragments. The resulting eccDNA was amplified using multiple displacement amplification before sonication into smaller fragments. The DNA was sequenced on a NovaSeq 6000, and the eccDNA was identified using the Circle_Finder algorithm. cfDNA, cell‐free DNA.

**Fig. 2:**
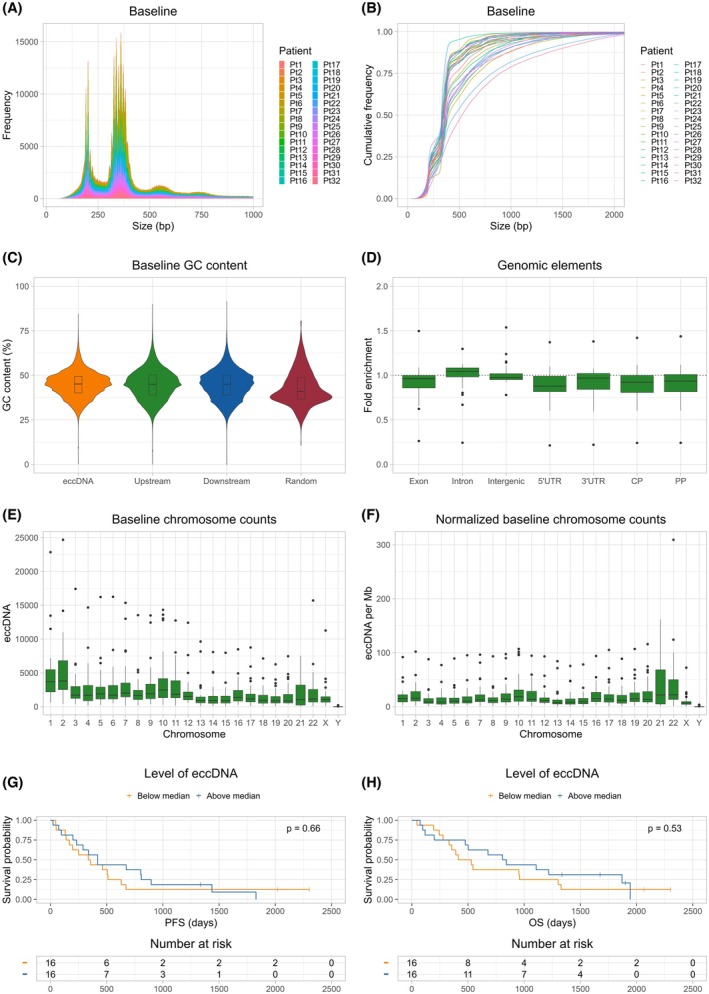
Characterization of baseline eccDNA. (A) Size distribution of baseline eccDNA for all 32 patients. The *P*‐values were calculated using the Wilcoxon rank‐sum test. (B) Cumulative frequency of eccDNA sizes at baseline for all 32 patients. (C) Violin plot showing the baseline GC content. The GC content was calculated for each baseline eccDNA along with the regions directly upstream and downstream for the eccDNA. One thousand random fragments from hg38 were used to represent the genomic average. The boxplots represent the median value and the interquartile range. (D) Fold enrichment of genomic elements present on the baseline eccDNA. The whiskers represent 1.5 times the interquartile range, with points outside this range shown as outliers. (E) Total baseline eccDNA distribution on the chromosomes. The *y*‐axis represents the absolute number of eccDNA identified on each chromosome. The whiskers represent 1.5 times the interquartile range, with points outside this range shown as outliers. (F) Normalized baseline eccDNA distribution on the chromosomes. The *y*‐axis represents the number of eccDNA identified per Mb for each chromosome. The whiskers represent 1.5 times the interquartile range, with points outside this range shown as outliers. (G) PFS stratified by the level of eccDNA at baseline. The *P*‐value was calculated using the log‐rank test. (H) OS stratified by the level of eccDNA at baseline. The *P*‐value was calculated using the log‐rank test. bp, base pair; CP, core promoter; eccDNA, extrachromosomal circular DNA; GC, guanine‐cytosine; Mb, megabase; OS, overall survival; PP, proximal promoter; Pt, patient; PFS, progression‐free survival; UTR, untranslated region.

The workflow for the isolation and identification of eccDNA is shown in Fig. 1 and outlined in the figure legend. We identified a median of 44 475 eccDNA per patient (range 6463–276 742 eccDNA) in the baseline plasma using the Circle_Finder algorithm along with a filter requiring the eccDNA to be mapped to chromosomes 1 to 22, X, or Y, and containing at least two junctional tags, which is the number of reads mapping to the eccDNA breakpoint. Information regarding sequencing metrics can be found in Table S2. No correlation between the number of reads and the identified number of both unfiltered and filtered eccDNA was found (Fig. S1). The eccDNA had a median size of 354.5 bp but with a wide range of sizes (33–244 825 049 bp). A distinctive bimodal size distribution was found with peaks corresponding to the size of one or two nucleosomes (Fig. 2A). Despite the wide range of eccDNA sizes, the clear majority of eccDNA was below 2000 bp (97.3–99.8% of the eccDNA) (Fig. 2B). To explore whether the base composition affected the generation of eccDNA, we analyzed the GC content of the eccDNA, as well as the GC content of the regions upstream and downstream of the eccDNA, with lengths corresponding to the length of the eccDNA. A similar GC content was found between the eccDNA and the upstream and downstream fragments (median [interquartile range (IQR)]: eccDNA: 45.1% [9.3], upstream: 45.0% [11.2], downstream: 45.0% [11.3]). The GC content of the eccDNA was compared with the GC content of one thousand random fragments from hg38 with a size corresponding to the median eccDNA size. The random fragments had a median GC content of 40.9% [IQR: 12.1], which was lower than what was observed for the eccDNA and surrounding regions (Fig. 2C). No enrichment of specific genomic elements was identified. The eccDNA was distributed throughout the exonic, intronic, and intergenic regions as expected by chance (Fig. 2D). The eccDNA was found on all chromosomes with the largest variation between patients on chromosomes 21 and 22 after normalization to chromosome size (Fig. 2E,F). No association between the baseline level of eccDNA and survival was found, neither in terms of PFS (345.5 vs. 420 days, HR = 0.85 [95% confidence interval (CI): 0.40–1.8], *P* = 0.66) nor OS (472 vs. 825 days, HR = 0.78 [95% CI: 0.36–1.7], p = 0.53) (Fig. 2G,H).

### 3.3 Characterization of eccDNA across samples and time points

Besides baseline samples, the patients had a response plasma sample collected after a median of 28 days (range 21–90 days) of osimertinib treatment. Two patients had no response sample available, resulting in a cohort of 30 patients having a response sample. At the control scan conducted closest to the time of response sample collection, 12 patients (40.0%) showed a partial response, 12 patients (40.0%) had stable disease, three patients (10.0%) exhibited progressive disease, and three patients (10.0%) did not have a scan performed near the time of response sample collection.

**Fig. 3:**
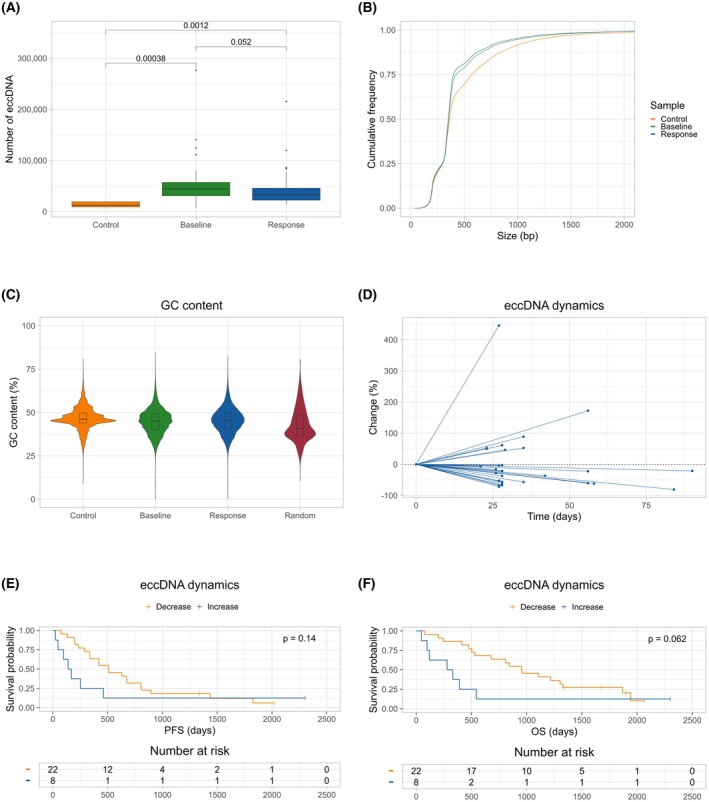
Characterization of eccDNA in cancer patients and healthy individuals. (A) Number of eccDNA in the control, baseline, and response samples. The whiskers represent 1.5 times the interquartile range, with points outside this range shown as outliers. (B) Cumulative frequency of eccDNA sizes for the control, baseline, and response samples. (C) Violin plot showing the GC content across sample types. GC content was calculated for each eccDNA in the control, baseline, and response samples. One thousand random fragments from hg38 were used to represent the genomic average. The boxplots represent the median value and the interquartile range. (D) Percentage change in the number of eccDNA from baseline to the response sample. The *x*‐axis represents the number of days from treatment initiation to response sample collection. Each line represents an individual patient. Lines from patients with similar percentage changes may overlap and limit visual distinguishability between individual patient lines. (E) PFS stratified by the dynamics in eccDNA from baseline to response. The *P*‐value was calculated using the log‐rank test. (F) OS stratified by the dynamics in eccDNA from baseline to response. The *P*‐value was calculated using the log‐rank test. bp, base pair; eccDNA, extrachromosomal circular DNA; GC, guanine‐cytosine; OS, overall survival; PFS, progression‐free survival.

A median of 32 691 eccDNA was identified per patient (range 13 796–215 385) in the response samples. There was no statistically significant difference in the number of eccDNA identified in the baseline and response samples (*P* = 0.052); however, the baseline samples exhibited a numerically higher number of eccDNA compared with the response samples (median: 44 475 vs 32 691) (Fig. 3A). As a control, we sequenced eccDNA‐enriched DNA from five healthy individuals. eccDNA was identified in all control samples with a median of 12 078 eccDNA per control sample (range 7849–20 757). The number of eccDNA was significantly lower in the control samples than in the baseline (*P* < 0.001) and response samples (*P* = 0.0012) (Fig. 3A). The eccDNA identified in the baseline and response samples from the cancer patients and the control samples from the healthy individuals displayed a similar size distribution (Fig. S2). The cumulative size frequency of the three sample types was comparable; however, the control samples displayed a lower fraction of eccDNA with a size below 1000 bp (Fig. 3B). However, this is attributed to a single control sample (C1) having a lower proportion of small eccDNA (Fig. S2E). The GC content of the baseline, response, and control eccDNA was comparable; however, with the control eccDNA displaying a narrower IQR [median (IQR): eccDNA: 45.1% (9.3), response: 45.5% (9.2), control: 46.2% (6.1)] (Fig. 3C). To discern whether the eccDNA could be generated by microhomology‐mediated end‐joining, we investigated whether 2‐ to 15‐bp direct repeats could be identified directly upstream and downstream of the eccDNA breakpoint. Microhomology was identified in a median of 16.1% of baseline eccDNA (range 9.4–28.2%), 14.0% of response eccDNA (range 9.4–27.1%), and in 20.2% of control eccDNA (range 11.7–28.8%). No statistically significant difference was observed between degrees of microhomology in the three sample types (Fig. S3).

Of the patients, 22/30 (73.3%) experienced a decrease in the number of eccDNA following treatment initiation, with a median decrease of 44.7% (2.3–80.8%). The eight patients (26.7%) experiencing an increase in the number of eccDNA had a median increase of 56.9% (46.4–445.6%) (Fig. 3D). A numerically longer PFS [508.5 vs 154 days, HR = 0.53 (95% CI: 0.23–1.4), *P* = 0.14] and OS [955.5 vs 305.5 days, HR = 0.43 (95% CI: 0.18–1.1), *P* = 0.062] were observed in patients with a decreasing number of eccDNA, although they were not statistically significant (Fig. 3E,F). Similarly, a numerically longer PFS and OS were observed for patients with a below‐median level of response eccDNA, but they were not statistically significant [PFS: 630 vs 200 days, HR = 0.48 (95% CI: 0.22–1.0), *P* = 0.053, OS: 1107 vs 332 days, HR = 0.52 (95% CI: 0.23–1.2), *P* = 0.10] (Fig. S4).

### 3.4 *EGFR* ‐overlapping eccDNA

**Fig. 4:**
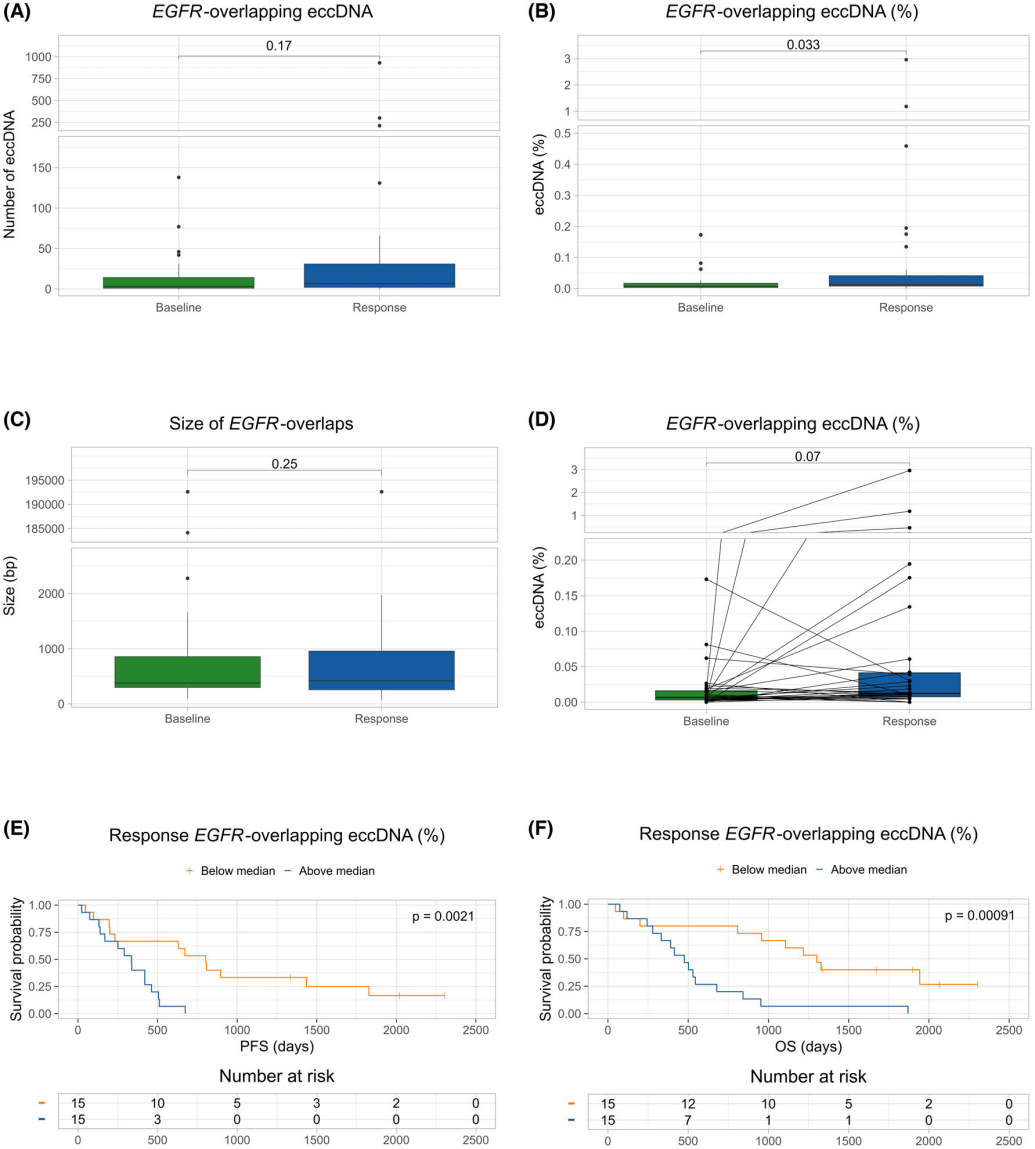
Characterization of *EGFR*‐overlapping extrachromosomal circular DNA (eccDNA). (A) Number of *EGFR*‐overlapping eccDNA in the baseline and response samples. The whiskers represent 1.5 times the interquartile range, with points outside this range shown as outliers. The *P*‐value was calculated using the Wilcoxon rank‐sum test. (B) The proportion of *EGFR*‐overlapping eccDNA out of all identified eccDNA in the baseline and response samples. The whiskers represent 1.5 times the interquartile range, with points outside this range shown as outliers. The *P*‐value was calculated using the Wilcoxon rank‐sum test. (C) The size of the *EGFR* fragment contained in the eccDNA overlaps for the baseline and response samples. The whiskers represent 1.5 times the interquartile range, with points outside this range shown as outliers. The p‐value was calculated using the Wilcoxon rank‐sum test. (D) Paired analysis showing the dynamics in the proportion of *EGFR*‐overlapping eccDNA in the baseline and response samples. The whiskers represent 1.5 times the interquartile range, with points outside this range shown as outliers. The *P*‐value was calculated using the paired Wilcoxon signed‐rank test. (E) PFS stratified by the level of *EGFR*‐overlapping eccDNA (%) at response. The *P*‐value was calculated using the log‐rank test. (F) OS stratified by the level of *EGFR*‐overlapping eccDNA (%) at response. The *P*‐value was calculated using the log‐rank test. bp, base pair; eccDNA, extrachromosomal circular DNA; EGFR, epidermal growth factor receptor; OS, overall survival; PFS, progression‐free survival.

All cancer patients in this study had a biopsy‐verified activating *EGFR* mutation before osimertinib treatment initiation. We investigated whether the identified eccDNA contained parts of the *EGFR* gene; these eccDNA are referred to as *EGFR‐*overlapping eccDNA. *EGFR*‐overlapping eccDNA was identified in 29/32 (90.6%) of the baseline samples (median 3, range 0–138), in 27/30 (90.0%) of the response samples (median 6.5, range 0–931), and in 3/5 (60.0%) of the control samples (median 1, range 0–179). No significant difference was found between the absolute number of *EGFR*‐overlapping eccDNA between baseline and response samples (*P* = 0.17) (Fig. 4A). To avoid bias from the differences in the total number of eccDNA identified per patient, the *EGFR*‐overlapping eccDNA number was normalized to the total number of eccDNA identified for each patient. This showed that the response samples have a significantly higher proportion of *EGFR*‐overlapping eccDNA than the baseline samples (*P* = 0.033) (Fig. 4B). The baseline samples had a median overlap of 378 bp (96–192 611), and the response samples had a median overlap of 419 bp (66–192 611) (*P* = 0.25) (Fig. 4C). Notably, we identified a significantly longer survival for patients with low levels of *EGFR*‐overlapping eccDNA (proportion below median) at response, both in terms of PFS [803 vs 336 days, HR = 0.24 (95% CI: 0.09–0.61), *P* = 0.002] and OS [1302 vs 476 days, HR = 0.23 (95% CI: 0.09–0.57), *P* < 0.001] (Fig. 4E,F). In contrast, we found no association between the level of *EGFR*‐overlapping eccDNA at baseline and PFS [463 vs 346.5, HR = 0.81 (95% CI: 0.38–1.7), *P* = 0.58] or OS [897.5 vs 489 days, HR = 0.68 (95% CI: 0.31–1.5), *P* = 0.32] (Fig. S5).

Following osimertinib treatment initiation, 11/30 patients (36.7%) experienced a decrease in the percentage of *EGFR*‐overlapping eccDNA (7.4–100.0% decrease), whereas 19 patients (63.3%) experienced an increase (27.8–23540.6% increase) (Fig. 4D). No association between dynamics in *EGFR*‐overlapping eccDNA and PFS [672 vs 336 days, HR = 0.72 (95% CI: 0.31–1.6), *P* = 0.41] or OS [958 vs 545 days, HR = 0.70 (95% CI: 0.28–1.6), *P* = 0.40] was found (Fig. S5).

## 4 Discussion

In this study, we isolated and analyzed plasma eccDNA from 32 patients with *EGFR*‐mutated advanced NSCLC both before and during treatment with osimertinib. We demonstrated that eccDNA can be identified in both the baseline and response plasma samples, as well as in plasma samples from healthy individuals. We showed that some eccDNA molecules will contain part of or the entire *EGFR* gene. Patients with high levels of *EGFR‐*overlapping eccDNA after a median of 4 weeks of osimertinib treatment had shorter PFS and OS compared to those with lower levels.

We observed that eccDNA was common in GC‐rich regions in both cancer and healthy control samples but was not enriched on any chromosomes. This is similar to studies investigating eccDNA in both tissue and plasma [7, 11, 17, 19, 20]. Additionally, our study observed a size distribution with a distinct bimodal pattern of eccDNA, consistent with findings from studies on plasma eccDNA in cancer patients and pregnant women [19, 20]. The bimodal peaks correspond to the size of one or two nucleosomes, including the DNA linker, indicating that the eccDNA remains wrapped around the histone octamer. We speculate that the size of one or two nucleosomes represents the most stable circular structure for plasma eccDNA. The dinucleosomal structure may be the preferred size because a mononucleosomal structure will have to overcome a high degree of torsional stiffness to ligate the two DNA ends [35, 36]. Larger eccDNA fragments could be more prone to cleavage with endonucleases due to a potential higher presence of cleavage sites. The dinucleosomal eccDNA molecule may represent an ideal size, where the two ends are in close proximity while retaining a certain level of flexibility, yet small enough to represent a stable structure. Furthermore, we observed a distinct pattern of smaller peaks at every 10 bp, corresponding to a turn of the DNA helix [37].

The clear majority of eccDNA identified in plasma was below 2000 bp. This finding is consistent with earlier studies on plasma eccDNA [17, 20]. The presence of only smaller eccDNA in plasma, in contrast to studies that identify eccDNA ranging from kilobases to megabases in tissue or cell lines, could be due to larger eccDNA being more unstable in plasma. Alternatively, fragmentation of larger tissue eccDNA before its release into the bloodstream could also be a factor. The release mechanism of eccDNA from cells is still not fully understood, but eccDNA may be released during apoptosis or necrosis, or it could potentially be subjected to active release, especially if plasma eccDNA has a regulatory function [13, 14]. A study by Sin et al. demonstrated that different nucleases might be responsible for the intra‐ and extracellular digestion of eccDNA and that digestion by different nucleases could result in distinct size distributions of eccDNA [38]. We observed no differences in size distribution between cancerous and healthy eccDNA, indicating a similar digestion profile. The subsequent ligation could be mediated by processes, such as microhomology‐mediated end‐joining, non‐homologous end‐joining, and homologous recombination [2]. We identified microhomology surrounding 9.4–28.8% of the eccDNA breakpoints, demonstrating a large degree of interpatient variation in microhomology levels. This suggests that microhomology‐mediated end‐joining may contribute to the formation of eccDNA, although it is not the sole mechanism involved.

We were able to detect eccDNA in all five healthy individuals, but in a significantly lower number than in both baseline and response samples from the NSCLC patients. The identified eccDNA had a similar size distribution and GC content as the cancer eccDNA. This indicates that the presence of eccDNA is not an inherent cancer characteristic, but the higher level of eccDNA in cancer patients underscores the potential of eccDNA as a biomarker for cancer diagnostics and screening. In mice and humans, eccDNA molecules have been reported to be common in somatic tissue, and it has been suggested that eccDNA may be a byproduct of deletions or DNA damage and could potentially play a biological role in healthy tissues as well as in cancer [10, 11, 17, 39].

Following a median of 4 weeks of treatment with osimertinib, we observed that almost three‐quarters of the patients experienced a decrease in the number of eccDNA. We observed a numerically longer PFS and OS for patients with a decreased level of eccDNA compared to those with an increased level of eccDNA, but they did not reach statistical significance. It is worth noting that only eight patients were included in the subgroup with an increase in eccDNA during treatment, and one of these eight patients (Pt10) had the longest survival of all patients (censored at day 2303). Thus, a larger study should be conducted to verify this result.

The majority of patients harbored *EGFR*‐overlapping eccDNA, which was also detected in three out of five healthy individuals. The response samples had the highest proportion of *EGFR*‐overlapping eccDNA. There was no clear trend in the dynamics of *EGFR*‐overlapping eccDNA, with 19 patients experiencing an increase in the percentage of *EGFR*‐overlapping eccDNA, whereas 11 patients experienced a decrease. Importantly, we found that patients with a below‐median proportion of *EGFR*‐overlapping eccDNA at the response time point had significantly longer PFS and OS than patients with a high proportion of *EGFR*‐overlapping eccDNA. We did not find any association with PFS and OS in terms of the level of *EGFR*‐overlapping eccDNA at baseline or the dynamics in *EGFR*‐overlapping eccDNA during treatment. Thus, in this cohort, neither the baseline level nor the dynamics of *EGFR*‐overlapping eccDNA were important for survival. In contrast, low levels of eccDNA containing part of the *EGFR* gene following treatment initiation were associated with longer PFS and OS. If the majority of *EGFR*‐overlapping eccDNA originates from cancer cells, it is plausible that effective treatment will lead to a reduction in cancer cells and thereby a lower proportion of eccDNA containing the *EGFR* gene. In contrast to this, Nathanson *et al*. proposed that the disappearance of mutant *EGFR* from eccDNA may be a mechanism of resistance to *EGFR*‐TKI treatment [12] However, the study was conducted on cancer cell lines investigating only mutant *EGFR* fragments on eccDNA, which may explain the discrepancy.

The main limitation of this study is the number of patients, which restricts their stratification into subgroups in survival analyses. However, this study is to our knowledge one of the largest investigating the presence of plasma eccDNA in a homogenous cancer patient cohort. Furthermore, this is the first research to explore the dynamics of plasma eccDNA in response to systemic cancer treatment. Our findings demonstrate that eccDNA is present in plasma, and can be isolated, sequenced, and detected using available techniques and workflows. We show that plasma eccDNA is upregulated in cancer patients compared with healthy individuals. Furthermore, we demonstrate that patients with low levels of *EGFR*‐overlapping eccDNA during treatment had significantly longer survival compared to those with higher levels. These findings open up a multitude of possibilities for further studies and underline the importance of investigating the potential function of plasma eccDNA in cancer, as well as its potential as a biomarker. This study was conducted in lung cancer patients, but the findings could be relevant for various types of cancer and even other non‐cancerous diseases.

## 5 Conclusion

Plasma eccDNA is present in both cancer patients and healthy individuals. In a cohort of *EGFR*‐mutated NSCLC patients, we observed that the majority of patients had eccDNA molecules containing part of or the entire *EGFR* gene. High levels of *EGFR*‐overlapping eccDNA during treatment were associated with poor outcomes, and eccDNA could serve as a biomarker for treatment efficacy.

## Conflict of interest

The authors declare no conflict of interest.

## Author contributions

SS and BSS contributed to the conceptualization. SS, SM, AD, and BSS contributed to the methodology. SS and SM contributed to the formal analysis. SS contributed to the investigation, visualization, and writing—original draft preparation. EBFE, PM, and BSS contributed to the resources. SM, EBFE, PM, and AD contributed to the data curation. SS, SM, EBFE, PM, AD, and BSS contributed to the writing—review and editing. PM, AD, and BSS contributed to the supervision. All authors have read and agreed to the final version of the manuscript.

## Supporting information


**Fig. S1.** Linear regression analyses with 95% prediction bands. (A) Correlation between the raw number of reads and unfiltered eccDNA. (B) Correlation between the trimmed number of reads and unfiltered eccDNA. (C) Correlation between the raw number of reads and filtered eccDNA. (D) Correlation between the trimmed number of reads and filtered eccDNA. eccDNA, extrachromosomal circular DNA.

**Fig. S2.** Size distribution profiles for the response and control samples. (A) Size distribution of response eccDNA for 30 patients. (B) Size distribution of control eccDNA for five healthy individuals. (C) Size distribution of control eccDNA for five healthy individuals with adjusted *y*‐axis. (D) Cumulative frequency of eccDNA sizes at response for 30 patients. (E) Cumulative frequency of eccDNA sizes for five healthy individuals. bp, base pair; Pt, patient.

**Fig. S3.** Microhomology for the control, baseline, and response samples. The *y*‐axis represents the percentage of eccDNA containing microhomology around the breakpoint. The *P*‐values were calculated using the Wilcoxon rank‐sum test. eccDNA, extrachromosomal circular DNA.

**Fig. S4.** Survival analysis for the level of eccDNA in response samples. (A) PFS stratified by the level of eccDNA at response. (B) OS stratified by the level of eccDNA at response. The *P*‐values were calculated using the log‐rank test. eccDNA, extrachromosomal circular DNA; OS, overall survival; PFS, progression‐free survival.

**Fig. S5.** Survival analysis for *EGFR*‐overlapping eccDNA. (A) PFS stratified by the dynamics of *EGFR*‐overlapping eccDNA from baseline to response. (B) OS stratified by the dynamics of *EGFR*‐overlapping eccDNA from baseline to response. (C) PFS stratified by the level of *EGFR*‐overlapping eccDNA (%) at baseline. (D) OS stratified by the level of *EGFR*‐overlapping eccDNA (%) at baseline. The *P*‐values were calculated using the log‐rank test. eccDNA, extrachromosomal circular DNA; EGFR, epidermal growth factor receptor; OS, overall survival; PFS, progression‐free survival.

**Table S1.** Baseline characteristics.

**Table S2.** Sequencing metrics.

## Data Availability

Research data are available upon reasonable request to the corresponding author.
