# Supplementary figures and images for: Plasma extrachromosomal circular DNA as a biomarker in EGFR‐targeted therapy of non‐small cell lung cancer
Source: Mol Oncol. 2025 Oct 30;20(4):1061–73. doi: 10.1002/1878-0261.70138 (PMC13060643; doi:10.1002/1878-0261.70138)

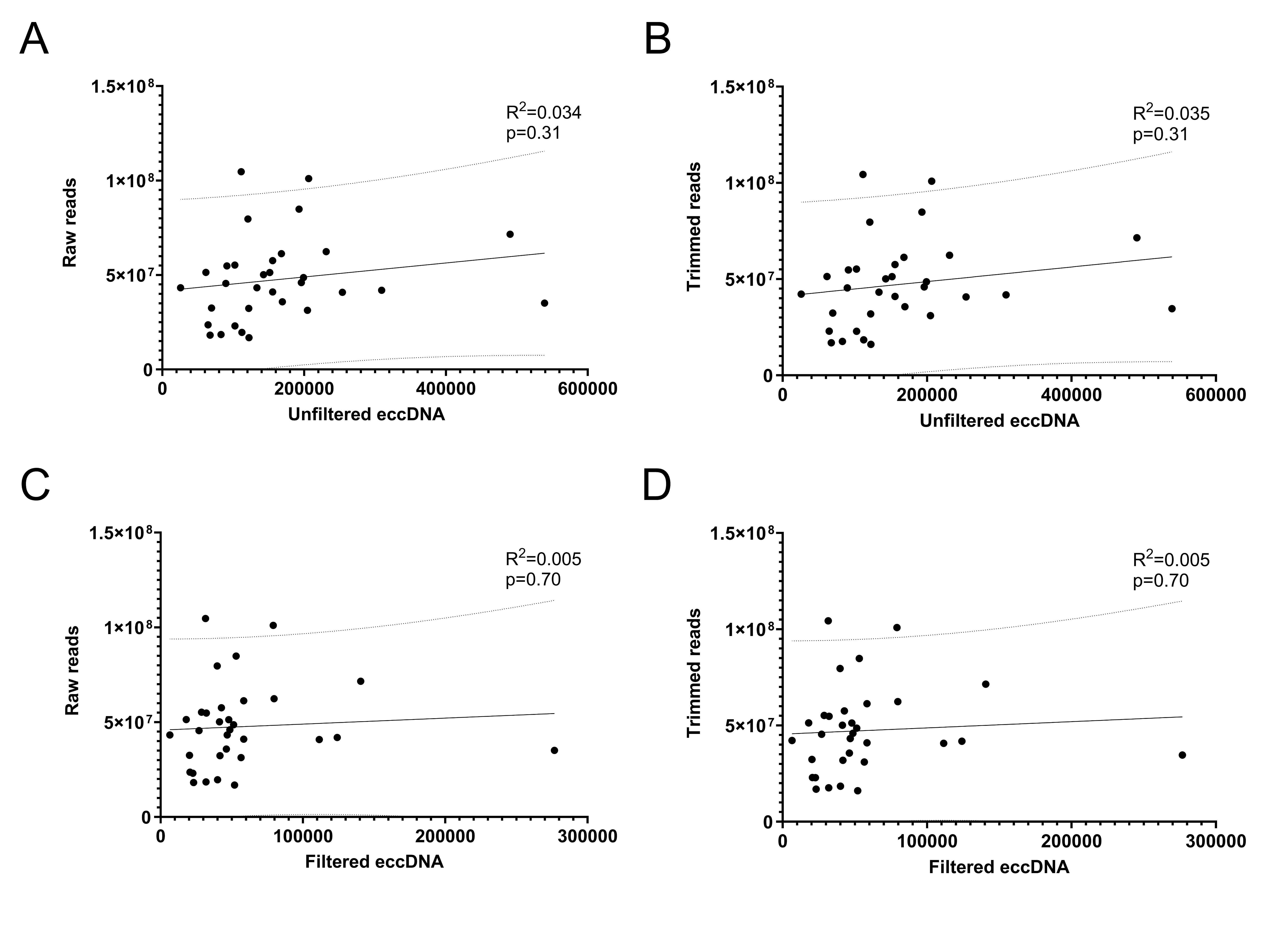

Supplement: Supplementary file 1 — Fig. S1. Linear regression analyses with 95% prediction bands. (A) Correlation between the raw number of reads and unfiltered eccDNA. (B) Correlation between the trimmed number of reads and unfiltered eccDNA. (C) Correlation between the raw number of reads and filtered eccDNA. (D) Correlation between the trimmed number of reads and filtered eccDNA. eccDNA, extrachromosomal circular DNA. [file MOL2-20-1061-s005.jpg]

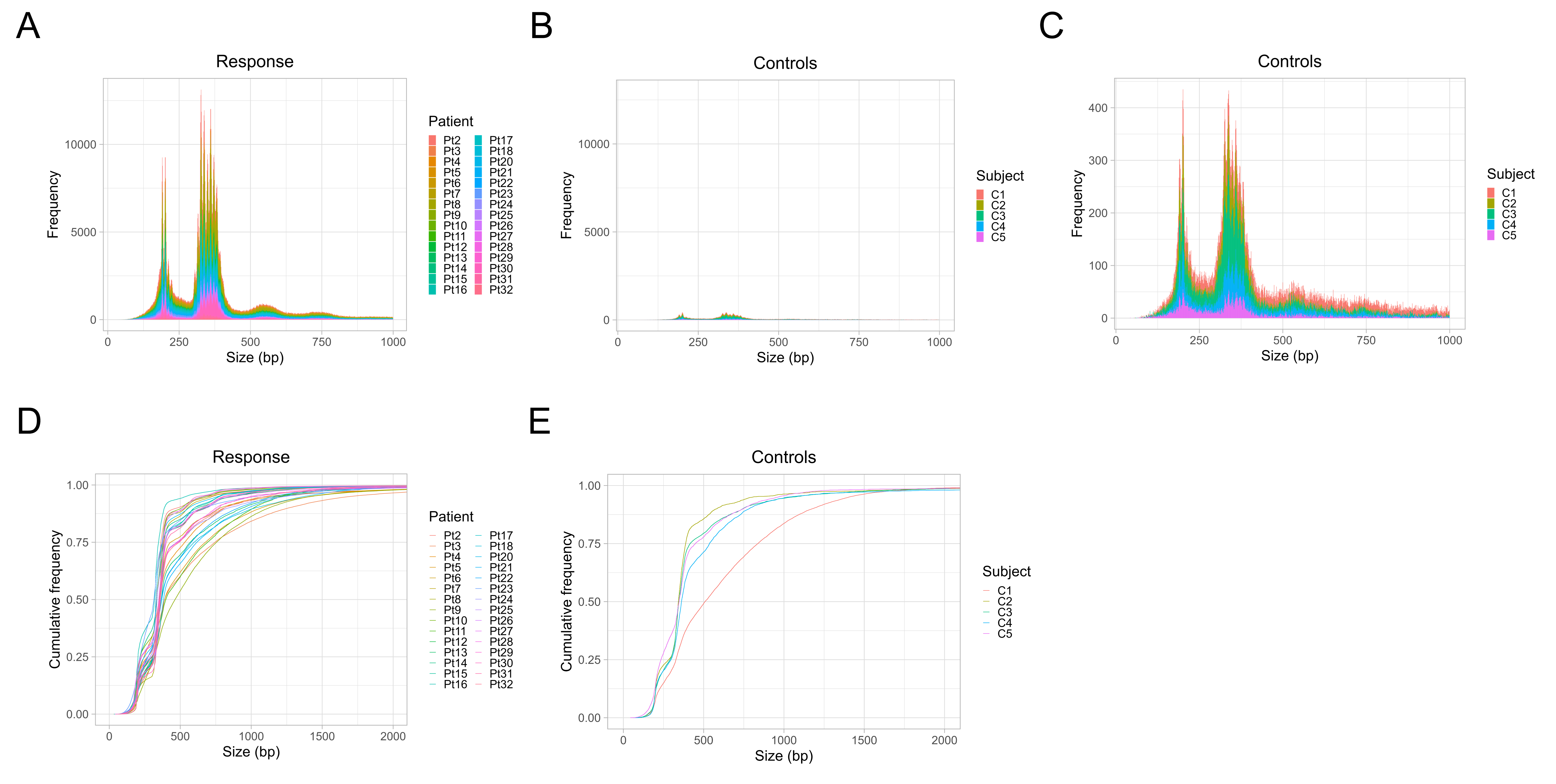

Supplement: Supplementary file 2 — Fig. S2. Size distribution profiles for the response and control samples. (A) Size distribution of response eccDNA for 30 patients. (B) Size distribution of control eccDNA for five healthy individuals. (C) Size distribution of control eccDNA for five healthy individuals with adjusted y‐axis. (D) Cumulative frequency of eccDNA sizes at response for 30 patients. (E) Cumulative frequency of eccDNA sizes for five healthy individuals. bp, base pair; Pt, patient. [file MOL2-20-1061-s007.png]

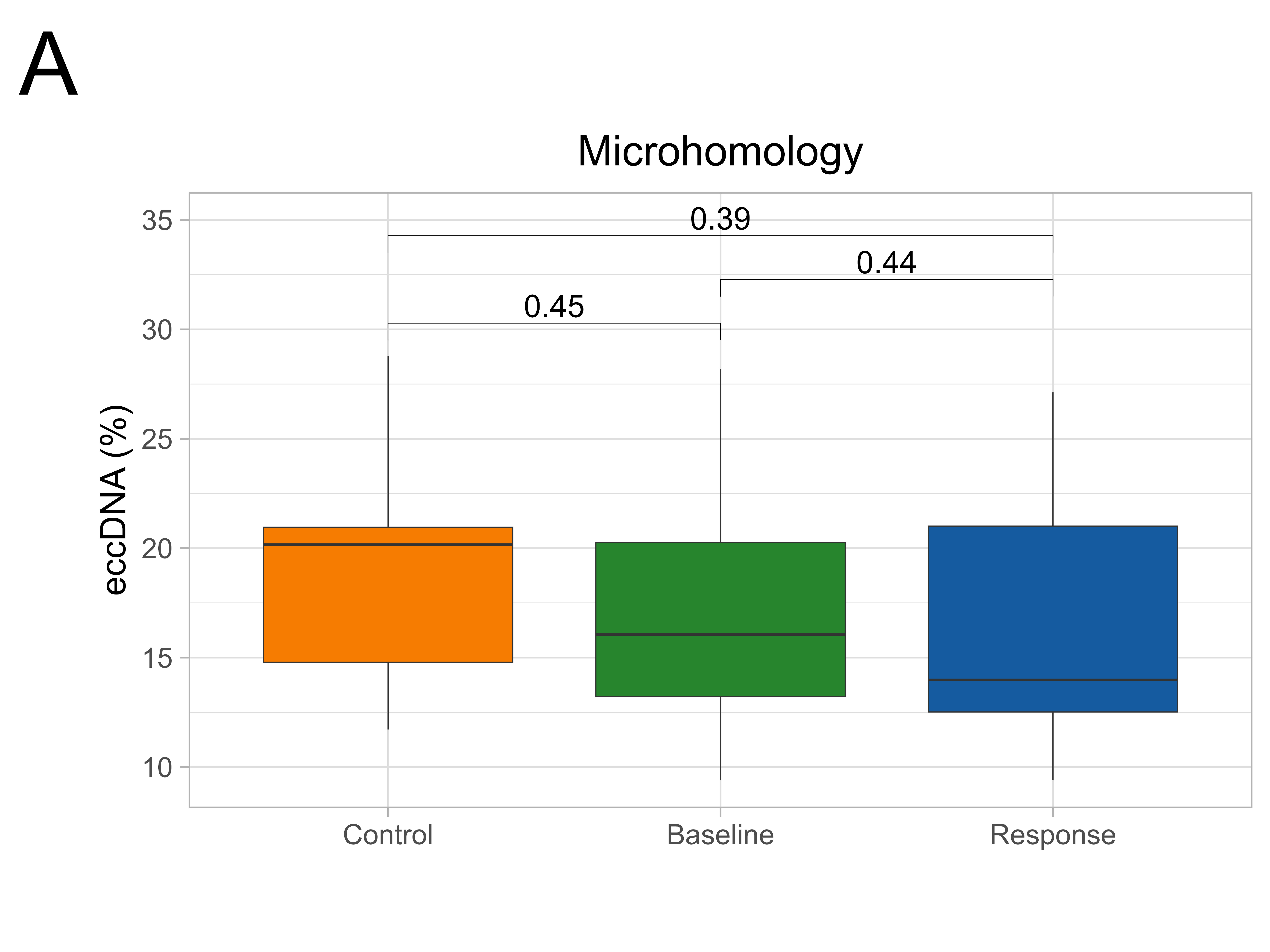

Supplement: Supplementary file 3 — Fig. S3. Microhomology for the control, baseline, and response samples. The y‐axis represents the percentage of eccDNA containing microhomology around the breakpoint. The P‐values were calculated using the Wilcoxon rank‐sum test. eccDNA, extrachromosomal circular DNA. [file MOL2-20-1061-s002.jpg]

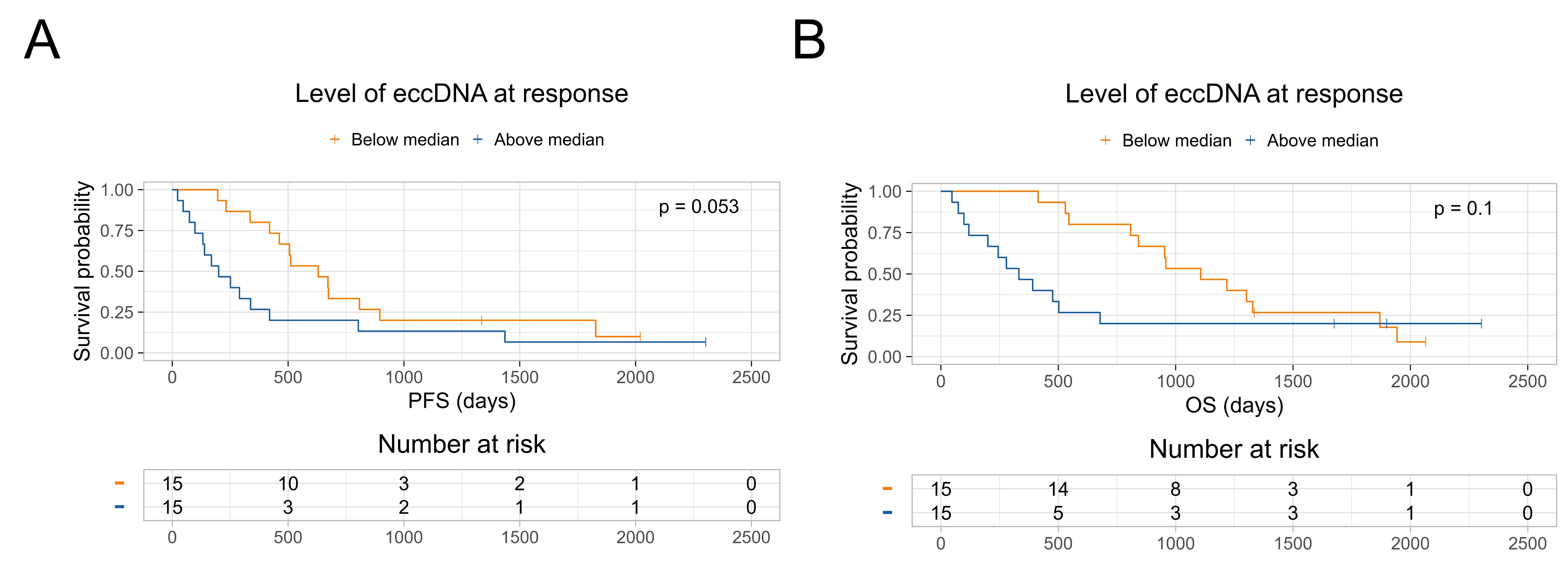

Supplement: Supplementary file 4 — Fig. S4. Survival analysis for the level of eccDNA in response samples. (A) PFS stratified by the level of eccDNA at response. (B) OS stratified by the level of eccDNA at response. The P‐values were calculated using the log‐rank test. eccDNA, extrachromosomal circular DNA; OS, overall survival; PFS, progression‐free survival. [file MOL2-20-1061-s004.jpg]

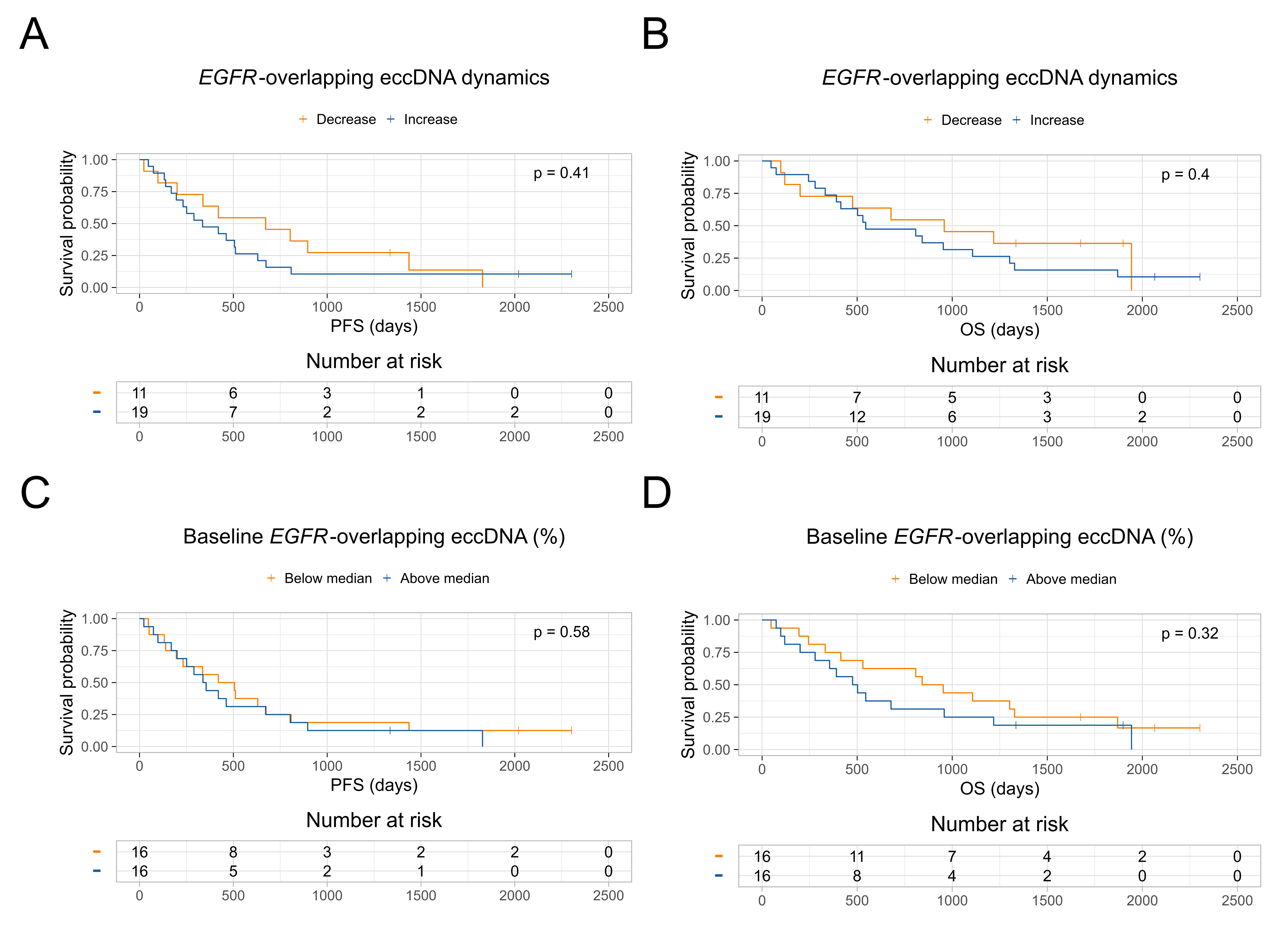

Supplement: Supplementary file 5 — Fig. S5. Survival analysis for EGFR‐overlapping eccDNA. (A) PFS stratified by the dynamics of EGFR‐overlapping eccDNA from baseline to response. (B) OS stratified by the dynamics of EGFR‐overlapping eccDNA from baseline to response. (C) PFS stratified by the level of EGFR‐overlapping eccDNA (%) at baseline. (D) OS stratified by the level of EGFR‐overlapping eccDNA (%) at baseline. The P‐values were calculated using the log‐rank test. eccDNA, extrachromosomal circular DNA; EGFR, epidermal growth factor receptor; OS, overall survival; PFS, progression‐free survival. [file MOL2-20-1061-s001.jpg]
