# Supplementary material for: Plasma extrachromosomal circular DNA as a biomarker in EGFR‐targeted therapy of non‐small cell lung cancer
Source: Mol Oncol. 2025 Oct 30;20(4):1061–73. doi: 10.1002/1878-0261.70138 (PMC13060643; doi:10.1002/1878-0261.70138)
Supplement: Supplementary file 6 — Table S1. Baseline characteristics. [file MOL2-20-1061-s006.docx]

Supplementary Table S1: Baseline characteristics

| **Characteristics** | **Patient cohort**  **(n = 32)** |
| --- | --- |
| **Age at treatment initiation (years)**  Median (range)  **Sex, n (%)**  Female  Male  **Histopathology, n (%)**  Adenocarcinoma  Not otherwise specified  **Metastasis stage, n (%)**  No metastasis  Pulmonary contralateral metastasis or pleural/pericardial effusion  Extrapulmonary metastasis  **Treatment line, n (%)**  First  Second  Third  **Smoking status, n (%)**  Active  Former  Never  ***EGFR* mutation, n (%)**  Ex19del^a^  L858R^b^  Ex20ins  **State of disease at baseline, n (%)**  PR  SD  PD  NA  **State of disease at response^c^, n (%)**  PR  SD  PD  NA | 67.5 (28-89)  24 (75.0)  8 (25.0)  31 (96.9)  1 (3.1)  6 (18.8)  8 (25.0)  18 (56.3)  3 (9.4)  26 (81.3)  3 (9.4)  2 (6.3)  18 (56.3)  12 (37.5)  21 (65.6)  10 (31.3)  1 (3.1)  0 (0.0)  6 (18.8)  23 (71.9)  3 (9.4)  12 (40.0)  12 (40.0)  3 (10.0)  3 (10.0) |

^a^4 patients had an additional T790M mutation

^b^3 patients had an additional T790M mutation

^c^30/32 patients had a response sample

NA, not answered; PD, progressive disease; PR, partial response; SD, stable disease
